# Body Mass Index, Smoking and Hypertensive Disorders during Pregnancy: A Population Based Case-Control Study

**DOI:** 10.1371/journal.pone.0152187

**Published:** 2016-03-24

**Authors:** Thuridur A. Gudnadóttir, Brian T. Bateman, Sonia Hernádez-Díaz, Miguel Angel Luque-Fernandez, Unnur Valdimarsdottir, Helga Zoega

**Affiliations:** 1 Faculty of Medicine, Centre of Public Health Sciences, University of Iceland, Reykjavík, Iceland; 2 Department of Anesthesia, Critical Care and Pain Medicine, Massachusetts General Hospital, Harvard Medical School, Boston, MA, United States of America; 3 Department of Medicine, Division of Pharmacoepidemiology and Pharmacoeconomics, Brigham and Women’s Hospital, Harvard Medical School, Boston, MA, United States of America; 4 Department of Epidemiology, Harvard T. H. Chan School of Public Health, Boston, MA, United States of America; Institute of Preventive Medicine, DENMARK

## Abstract

While obesity is an indicated risk factor for hypertensive disorders of pregnancy, smoking during pregnancy has been shown to be inversely associated with the development of preeclampsia and gestational hypertension. The purpose of this study was to investigate the combined effects of high body mass index and smoking on hypertensive disorders during pregnancy. This was a case-control study based on national registers, nested within all pregnancies in Iceland 1989–2004, resulting in birth at the Landspitali University Hospital. Cases (n = 500) were matched 1:2 with women without a hypertensive diagnosis who gave birth in the same year. Body mass index (kg/m^2^) was based on height and weight at 10–15 weeks of pregnancy. We used logistic regression models to calculate odds ratios and corresponding 95% confidence intervals as measures of association, adjusting for potential confounders and tested for additive and multiplicative interactions of body mass index and smoking. Women’s body mass index during early pregnancy was positively associated with each hypertensive outcome. Compared with normal weight women, the multivariable adjusted odds ratio for any hypertensive disorder was 1.8 (95% confidence interval, 1.3–2.3) for overweight women and 3.1 (95% confidence interval, 2.2–4.3) for obese women. The odds ratio for any hypertensive disorder with obesity was 3.9 (95% confidence interval 1.8–8.6) among smokers and 3.0 (95% confidence interval 2.1–4.3) among non-smokers. The effect estimates for hypertensive disorders with high body mass index appeared more pronounced among smokers than non-smokers, although the observed difference was not statistically significant. Our findings may help elucidate the complicated interplay of these lifestyle-related factors with the hypertensive disorders during pregnancy.

## Introduction

Hypertensive disorders, including chronic hypertension, gestational hypertension, and preeclampsia, occur in approximately 6–8% of all pregnancies [1] and are a significant source of maternal and fetal morbidity [2]. As the prevalence of advanced maternal age [3, 4] and obesity [5] increase among childbearing women in Western countries, hypertensive disorders are likely to become increasingly common obstetric conditions. Compared with offspring of normal pregnancies, offspring of preeclamptic pregnancies have a 1.5-to 2-fold increased risk of perinatal or infant mortality. Approximately a third of babies born after a preeclamptic pregnancy are growth restricted, and preeclampsia is responsible for 15% of all preterm births [6].

While obesity is a known risk factor for preeclampsia and other hypertensive disorders [7, 8], smoking during pregnancy has been shown to be inversely associated with the development of preeclampsia [9, 10]. Data are scarce on the potential interaction of these two lifestyle-related factors on these common pregnancy conditions [11]. To what extent maternal smoking and body mass index (BMI) interact in the development of preeclampsia and gestational hypertension is not well established. We therefore aimed to examine the association between BMI and hypertensive disorders during pregnancy, accounting for demographic and pregnancy related factors, as well as to assess the combined effect of BMI and smoking on hypertensive disorders among pregnant women.

## Materials and Methods

### Study design

We conducted a case-control study nested within all pregnancies in Iceland 1989–2004, which resulted in birth at the Landspitali University Hospital. The study was based on data from the National Medical Birth Registry and maternity records linked via women’s unique personal identification number.

Health care is publicly funded and accessible to everyone in Iceland. Prenatal care is offered in a uniform manner at local primary care clinics every two to four weeks of pregnancy (10 visits for nulliparous women and 7 visits for others), with the first prenatal visit occurring between pregnancy weeks 10 and 15. Women who need specialized care receive more follow up [12]. The Landspitali University Hospital provides secondary and tertiary services for the whole of Iceland. It covered approximately 2900 births annually, 45000 in total, during the study period [13, 14], mainly including mothers residing in the capital area (Table 1), as well deliveries among mothers in need of specialized medical care.

**Table 1:** Demographic and Pregnancy Characteristics of Cases and Controls^*^.

	Total N (%)	Cases n (%)	Controls n (%)	p-value
**Total**	1445 (100)	483 (100)	962 (100)	
**Age, years**	** **			
Mean (SD)	28.4 (± 5.9)	28.1 (± 6.3)	28.5 (± 5.7)	
< 25	409 (28.3)	157 (32.5)	252 (26.2)	0.008
25–34	793 (54.9)	238 (49.3)	555 (57.7)	
≥ 35	243 (16.8)	88 (18.2)	155 (16.1)	
**Nationality**				
Icelandic	1410 (97.6)	469 (97.1)	941 (97.8)	0.45
Other	32 (2.2)	13 (2.7)	19 (2.0)	
Missing	3 (0.2)	1 (0.2)	2 (0.2)	
**Residency**	** **			
capital area	1087 (75.2)	355 (73.5)	732 (76.1)	0.3
non capital area	257 (17.8)	105 (21.7)	152 (15.8)	
Missing	101 (7.0)	23 (4.8)	78 (8.1)	
**Cohabitation**	** **			
lives with other parent	1238 (85.7)	412 (85.3)	826 (85.9)	0.81
Single	205 (14.2)	70 (14.5)	135 (14.0)	
Missing	2 (0.1)	1 (0.2)	1 (0.1)	
**Working status**	** **			
paid work	1046 (72.4)	350 (72.5)	696 (72.3)	0.12
homemaker, not working, disability	28 (1.9)	8 (1.7)	20 (2.1)	
student	197 (13.6)	77 (15.9)	120 (12.5)	
Other	174 (12.0)	48 (9.9)	126 (13.1)	
**Parity, n**	** **			
0	659 (45.6)	285 (59.0)	374 (38.9)	<0.0001
1	436 (30.2)	102 (21.1)	334 (34.7)	
≥ 2	350 (24.2)	96 (19.9)	254 (26.4)	
**Multiple gestation**				
No	1348 (93.3)	437 (90.5)	911 (94.7)	0.004
Yes	97 (6.7)	(9.5)	51 (5.3)	

Abbreviations: HTD, hypertensive disorder of pregnancy; p-value, Fisher Exact Test for difference in proportions between cases and controls, SD, standard deviation.

* Cases and controls gave birth in the same calendar year. All cases and controls are restricted to women without any registered diabetes during pregnancy.

### Ascertainment of cases and controls

As cases we randomly selected a total of 500 women from the electronic National Medical Birth Registry with any registered hypertensive disorder during pregnancy (International Classification of Disease, 10^th^ Revision [ICD-10] codes, O10-16) [15] and no diagnosis of diabetes (ICD-10 codes, O24, E08-13). The population was restricted to women without any registered diagnoses of diabetes in light of evolving clinical guidelines and non-uniform screening of gestational diabetes during the study period [16]. Hypertensive disorders during pregnancy were further categorized as: *pre-existing hypertension* (O10), *gestational hypertension* (O13, O16) and *preeclampsia* (O14, O15, O11). Preeclampsia superimposed on pre-existing hypertension (O11) was included with preeclampsia, as only four such cases were identified. No cases of gestational edema and proteinuria without hypertension (O12) were identified. According to the diagnostic criteria [17], *pre-existing hypertension* is defined as hypertension that is present and observable before pregnancy or that is diagnosed before the 20th week of pregnancy; *gestational hypertension* and *preeclampsia* occur after pregnancy week 20.

As controls we randomly selected a total of 1000 women from the Medical Birth Registry, who had no registered diagnosis of a hypertensive disorder or diabetes during pregnancy and gave birth in the same year as each case (1 case: 2 controls). When selecting the number of cases and controls we assumed an odds ratio of at least 2.0 for any hypertensive disorder and 80% power.

### Measures of body mass index and smoking

Information on measured weight, height and self-reported smoking behavior during pregnancy was manually retrieved from maternity records. Weight (kg) and height (m) were measured at the first prenatal visit, which occurred on average at pregnancy week 13.2 (median, 13.8 weeks; interquartile range, 12.0–15.3 weeks) in the study population. Body mass index (BMI) was defined in accordance with international standards [18] as kg/m^2^ and used as a continuous and a categorical variable: *underweight* (BMI < 18.5), *normal weight* (BMI 18.5–24.9), *overweight* (BMI 25.0–29.9), *obese* (BMI ≥ 30.0). BMI was available for 1445 (96.3%) of the study population, i.e. 483 (96.4%) of cases and 962 (96.3%) of controls.

Smoking behavior was self-reported at first prenatal visit and categorized as: *non-smoker*, *smoker* and *discontinued smoker* (smoked during early pregnancy but quit). Of the 1445 women with known BMI, 77.8% (n = 1124) reported no smoking, 17.9% (n = 259) reported smoking during pregnancy, another 2.8% (n = 41) discontinued smoking during pregnancy, and 1.5% (n = 21) had an unknown smoking status. In the analyses we excluded discontinued smokers because of uncertainty of when exactly smoking had been discontinued. Information on quantity or type of smoking was not available.

### Covariates

Additionally, we obtained information on women’s demographic and pregnancy related characteristics from the National Medical Birth Registry, including maternal age at delivery, nationality, residency, working status and cohabitation with other parent, parity, multiple gestation and gestational length. Other registered maternal conditions, such as renal diseases (ICD-10 codes N00-N99, Q61, O27.1), collagen vascular diseases (ICD-10 codes L93, L94) and endocrine disorders (ICD-10 codes E00-E90) were assessed but not used as covariates because of too few observations.

### Data analysis

Of 1500 unique personal identification numbers extracted, records were incomplete or unavailable for three women and data on BMI missing for another 52 women; resulting in 1445 women (483 cases, 962 controls) available for the main data analysis. Analyses in which smoking status was included contained a total of 1383 women after exclusion of those with missing values (n = 21) and discontinued smoking (n = 41).

We first calculated the crude odds, odds ratios (OR) and corresponding 95% confidence intervals (CI), as measures of association of hypertensive disorders during pregnancies (any; pre-existing hypertension, gestational hypertension, preeclampsia) separately with BMI groups (normal weight, overweight, obese) and smoking status (no, yes). With logistic regression models we then simultaneously adjusted for all relevant covariates (maternal age at delivery, parity and multiple gestations) identified by backward selection and Yates continuity corrections [19]. Other available covariates did not affect the effect estimates, but in all instances calendar year was controlled for by the study design, i.e. matching on year of birth. As the matching criteria was wide, i.e. the only matching condition being that cases and controls gave birth during the same calendar year, we chose not to conduct conditional regression models. We tested additive and multiplicative interactions of maternal smoking and overweight/obesity and estimated multiplicative joint effects for maternal smoking and overweight/obesity.

We performed sensitivity analyses: 1) To validate the registration of hypertensive outcomes in the Medical Birth Registry we extracted systolic (SBP) and diastolic blood pressure (DBP) levels, measured at first and last prenatal visits, from the maternity records of all cases and controls. S1 Fig demonstrates higher blood pressure values among cases than controls, especially at the last prenatal visit. (**S1 Fig. Mean Blood Pressure (mm Hg) by Diagnosis of Hypertensive Disorder during Pregnancy**). We then further restricted hypertensive cases to those that met the threshold of at least 140 mmHg SBP or 90 mmHg DBP at the last (preeclampsia, gestational hypertension) and first prenatal visit (pre-existing hypertension). Data on proteinuria in maternity records were not accessible to use for validation of the hypertensive outcomes. 2) To evaluate the effect of potential misclassification of BMI exposure because of weight gained in early pregnancy, we restricted all analyses to women who had their first prenatal visit before the end of pregnancy week 15.

All analyses were performed using R.2.7 (R Foundation for Statistical Computing, Vienna, Austria) statistical software for computing. The study was approved by the Icelandic National Bioethics Committee (VSNb2012040011/03.07) and the Data Protection Authority (2012050619AT/-). An informed consent from women in the study population was not obtained as all personal information was anonymized and de-identified prior to analysis.

## Results

Demographic and pregnancy characteristics of women with hypertensive disorders during pregnancy (cases) and controls (no hypertensive disorders) are detailed in Table 1.

The demographic characteristics of cases and controls were largely similar, but they differed in parity. Hypertensive cases were more likely than controls to be nulliparous (59% vs. 39%), and they were also slightly younger than the controls (28.1 vs. 28.5 years). The distribution of characteristics of women with known and unknown BMI values were also similar (S1 Table), except women with missing BMI were more likely to be non-Icelandic.

According to measured height and weight at around 13 weeks of pregnancy, 1.7% (n = 24) of the 1445 women were underweight, 57.4% (n = 829) were normal weight, 26.0% (n = 376) overweight and 14.9% (n = 216) were obese. We collapsed the underweight women with the normal weight group in all analyses.

The odds of being diagnosed with a hypertensive disorder during pregnancy were lower for smokers than non-smokers (13.9% vs. 21.2%); with a crude odds ratio of 0.60 (95% CI 0.44–0.82). Adjusting for parity, multiple gestation, maternal age or any other available covariate did not affect the effect estimate (OR_adjusted_ = 0.60, 95% CI 0.44–0.82). We found a reverse association of smoking with each separate hypertensive outcome but the OR magnitude differed; preeclampsia (OR_adjusted_ = 0.68, 95% CI 0.47–0.96), gestational hypertension (OR_adjusted_ = 0.31, 95% CI 0.13–0.75) and pre-existing hypertension (OR_adjusted_ = 0.53, 95% CI 0.26–0.97). The point estimates of the reverse associations diminished in magnitude with increasing BMI, for any hypertensive disorder and for preeclampsia as shown in S2 Table.

The associations between BMI and hypertensive disorder during pregnancy are detailed in Table 2.

**Table 2:** Association of Body Mass Index with Hypertensive Disorders during Pregnancy.

	Total N	Cases n (%)	Controls n (%)	Crude OR (95% Cl)	Adjusted OR^*^ (95% Cl)
**Any HTD**	1445	483 (100)	962 (100)		** **
normal weight (BMI <24.9)	853	231 (47.8)	622 (64.7)	1.0	1.0
overweight (BMI 25–29.9)	376	142 (29.4)	234 (24.3)	1.63 (1.26–2.11)	1.75 (1.34–2.29)
obese (BMI ≥30)	216	110 (22.8)	106 (11.0)	2.79 (2.05–3.80)	3.08 (2.24–4.25)
**Preeclampsia**	1275	313	962		
normal weight (BMI <24.9)	783	161 (51.4)	622 (64.7)	1.0	1.0
overweight (BMI 25–29.9)	327	93 (29.7)	234 (24.3)	1.54 (1.14–2.06)	1.72 (1.26–2.34)
obese (BMI ≥30)	165	59 (18.9)	106 (11.0)	2.15 (1.49–3.08)	2.37 (1.62–3.46)
**Gestational hypertension**	1037	75	962		
normal weight (BMI <24.9)	657	35 (46.7)	625 (64.7)	1.0	1.0
overweight (BMI 25–29.9)	257	23 (30.7)	234 (24.3)	1.75 (1.00–3.01)	1.77 (1.00–3.09)
obese (BMI ≥30)	123	17 (22.7)	106 (11.0)	2.86 (1.51–5.23)	2.96 (1.55–5.47)
**Pre-existing hypertension**	1057	95	962		
normal weight (BMI <24.9)	657	35 (36.8)	622 (64.7)	1.0	1.0
overweight (BMI 25–29.9)	260	26 (27.4)	234 (24.3)	1.98 (1.15–3.35)	1.79 (1.04–3.06)
obese (BMI ≥30)	140	34 (35.8)	106 (11.0)	5.68 (3.38–9.55)	4.42 (3.20–9.21)

Abbreviations: BMI, body mass index; CI, confidence interval; HTD, hypertensive disorder of pregnancy; OR, odds ratio.

^*^ Adjusted for parity, multiple gestation and maternal age. Cases and controls gave birth in the same calendar year. All cases and controls are restricted to women without any registered diabetes during pregnancy.

Compared with normal weight women, both overweight and obese women had higher odds of any hypertensive disorders during pregnancy. The crude odds ratio for any hypertensive disorder was 1.63 (95% Cl 1.26–2.11) for overweight women and 2.79 (95% Cl 2.05–3.80) for obese women. Adjusting for parity, multiple gestation and maternal age amplified positive effect estimates somewhat. We found positive associations of BMI with each separate hypertensive outcome (preeclampsia, gestational hypertension and pre-existing hypertension). In comparison to normal weight women, the odds of preeclampsia and gestational hypertension increased by about 1.7-fold for overweight women and 2.4- to 3-fold for obese women, with effect estimates slightly higher when adjusting for parity, multiple gestation and maternal age. The adjusted odds ratios were highest for pre-existing hypertension; 1.79 and 4.42 among overweight and obese women, respectively, compared with normal weight women. Only maternal age seemed to affect the association between BMI and pre-existing hypertension, by slightly attenuating the estimates.

Table 3 demonstrates the estimated joint effect of overweight/obesity and smoking on hypertensive disorders during pregnancy.

**Table 3:** Association of Body Mass Index with Hypertensive Disorders during Pregnancy Stratified by Smoking Status.

	Non-smokers				Smokers			
	Total N	Cases n (%)	Controls n (%)	Adjusted OR^*^ (95% Cl)	Total N	Cases n (%)	Controls n (%)	Adjusted OR^*^ (95% Cl)
**Any HTD**	1124	402 (100)	722 (100)		259	65 (100)	194 (100)	
Normal weight (BMI <24.9)	669	199 (49.5)	470 (65.1)	1.0	148	26 (40.0)	122 (62.9)	1.0
Overweight (BMI25-29.9)	289	116 (28.9)	173 (24.0)	1.78 (1.31–2.42)	73	22 (33.8)	51 (26.3)	2.04 (1.05–3.96)
Obese (BMI >30)	166	87 (21.6)	79 (10.9)	2.98 (2.07–4.31)	38	17 (26.2)	21 (10.8)	3.91 (1.78–8.59)
**Preeclampsia**	975	253	722		242	48	194	
Normal weight (BMI <24.9)	607	137 (54.2)	470 (65.1)	1.0	141	19 (39.6)	122 (62.9)	1.0
Overweight (BMI25-29.9)	245	72 (28.5)	173 (24.0)	1.68 (1.18–2.40)	69	18 (37.5)	51 (26.3)	2.29 (1.09–4.79)
Obese (BMI >30)	123	44 (17.3)	79 (10.9)	2.19 (1.41–3.41)	32	11 (22.9)	21 (10.8)	3.41 (1.38–8.45)
**Gestational hypertension**	789	67	722		200	6	194	
Normal weight (BMI <24.9)	503	33 (49.3)	470 (65.1)	1.0	124	2 (33.3)	122 (62.9)	1.0
Overweight (BMI25-29.9)	193	20 (29.9)	173 (24.0)	1.75 (0.96–3.21)	54	3 (50)	51 (26.3)	3.74 (0.58–23.91)
Obese (BMI >30)	93	14 (20.9)	79 (10.9)	2.73 (1.37–5.46)	22	1 (16.7)	21 (10.8)	3.18 (0.26–38.44)
**Pre-existing hypertension**	804	82	722		205	11	194	
Normal weight (BMI <24.9)	499	29 (35.4)	470 (65.1)	1.0	127	5 (45.5)	122 (62.9)	1.0
Overweight (BMI25-29.9)	197	24 (29.2)	173 (24.0)	1.99 (1.11–3.58)	52	1 (9)	51 (26.3)	0.52 (0.06–4.64)
Obese (BMI >30)	108	29 (35.4)	79 (10.9)	5.87 (3.37–10.56)	26	5 (45.5)	21 (10.8)	6.28 (1.62–24.41)

Abbreviations: BMI, body mass index; CI, confidence interval; HTD, hypertensive disorder of pregnancy; OR, odds ratio.

* Adjusted for parity, multiple gestation and maternal age. Cases and controls gave birth in the same calendar year. All cases and controls are restricted to women without any registered diabetes during pregnancy.

The odds ratio for any hypertensive disorder was 3.91 (95% Cl 1.78–8.59) among smokers and 2.98 (95% Cl 2.07–4.31) among non-smokers, when comparing obese with normal weight women. The effect estimates stratified by smoking status were of similar magnitude for preeclampsia, but we lacked statistical power to meaningfully evaluate the association of BMI with gestational hypertension and pre-existing hypertension. Neither additive nor multiplicative interactions of maternal smoking and overweight/obesity were significant. We found a significant joint effect in a multiplicative scale for maternal smoking and obesity for any hypertension (p-value = 0.025), preexisting hypertension (p-value <0.001) but not for preeclampsia (p-value = 0.077).

Analyzing BMI as a continuous independent variable did not significantly alter the results of the main analysis associations demonstrated in Tables 2 and 3. Restricting all analyses to women with the first prenatal visit before end of pregnancy week 15 did not significantly alter the observed effect estimates. Restricting cases to women with the specified blood pressure criteria led to increased OR magnitudes for the associations of BMI with preeclampsia and gestational hypertension, but attenuated OR magnitudes for pre-existing hypertension (data not shown).

## Discussion

The results of this population based case-control study indicate that overweight and obese women are, compared with women of normal weight, at increased risk of hypertensive disorders during pregnancy, including preeclampsia and gestational hypertension. In our data the risk increased appeared to be amplified in women who smoke. Conversely, smoking alone appears to attenuate the risk of hypertensive disorders during pregnancy. These findings may help elucidate the complicated interplay of these lifestyle-related factors with the hypertensive disorders.

The risk increase we observed for hypertensive disorders during pregnancy was nearly 2-fold among overweight women and 3-fold among obese women when compared with normal weight women. A dose-dependent relation of BMI was apparent with each separate hypertensive outcome, i.e. preeclampsia, pre-existing and gestational hypertension. This is consistent with previous results found among pregnant women, although most have focused on the relationship with preeclampsia [20, 21]. But as Bateman et al. [22] demonstrated, using nationally representative data from NHANES, obesity is an independent risk factor for hypertension among all women of reproductive age, with prevalence increasing in near linear fashion with BMI.

Consistent with the elevated risk for preeclampsia observed among overweight (OR = 1.7) and obese (OR = 2.4) women in our data, Bodnar et al. [20] also found that pre-pregnancy BMI had a dose-dependent relation with preeclampsia, with a triple risk among women with a BMI of 30 compared with women who had a BMI of 21. These findings are in line with the results of a systematic review from 2003, including 13 cohort studies, that the risk of developing preeclampsia doubled for every 5–7 kg/m^2^ increase in BMI [23]. Most previous studies assessing BMI and gestational hypertension have analyzed it as a combined outcome with preeclampsia [20, 24]. As these two hypertensive conditions have been shown to share many risk factors [25], their similar association with BMI as demonstrated in our data is not of surprise.

Pre-existing hypertension and preeclampsia/gestational hypertension are, on the other hand, viewed as independent entities [17]. While the latter generally occur after week 20 of pregnancy, pre-existing hypertension is by definition detected earlier. Several studies have, however, demonstrated these different entities to share at least one risk factor, namely obesity [22, 26]. But as BMI and smoking-status were measured after the onset of pre-existing hypertension in our data, we cannot ascertain these as definite causal risk factors for pre-existing hypertension, even though effect estimates indicate associations thereof. Advanced age has also been considered in previous studies a risk factor for pre-existing hypertension [22]. After adjusting for age in our data the positive relationship between overweight/obesity and pre-existing hypertension remained, although slightly attenuated, which could reflect an independent effects of age or the duration of obesity or smoking habits on the outcome.

In line with previous findings [27–29], our data showed an inverse association between smoking and preeclampsia/gestational hypertension, primarily in women of normal weight. Conclusive results regarding potential effect modification of BMI on this association between smoking and hypertensive disorders can, however, not be drawn from our data due to lack of statistical power. Although non-conclusive, underlying biologic pathways for this inverse association have been hypothesized; such as the role of circulating angiogenic proteins [30], which could be released through the combustion of tobacco products, e.g. carbon monoxide [29]. Recently it has also been hypothesized that this inverse association with smoking may be due to miscarriage, or preterm delivery, of pregnancies with poor placentation due to smoking, i.e. smokers that would have developed hypertension or preeclampsia had they completed 40 weeks [31, 32]. In our data, cases, i.e. women with hypertensive disorders, were more likely than controls to give births preterm.

But how does smoking affect the observed risk increase among overweight and obese women for hypertensive disorders? Although our results indicated that the increased risk of hypertensive disorders with overweight/obesity might be higher among smokers than non-smokers, observations based on a larger number of pregnancies are needed to draw a definite conclusion on this. Previous studies have shown somewhat conflicting results on the combined association of BMI and smoking on the hypertensive outcomes during pregnancy. While, very few studies have examined smoking as an effect modifier, some have studied how BMI affects the association between smoking and hypertensive disorders. Stone et al. [33] suggested in a retrospective study based on data from birth certificates, that smoking decreased the risk for preeclampsia regardless of women pre-pregnancy BMI. While on the other hand, Ness et al. [11] showed in a large, prospective cohort study that the inverse association between preeclampsia and smoking was eliminated if a woman was overweight or obese. However, as their study data were based on 7,757 pregnancies from over five decades ago, i.e. 1959 to 1965, obese and overweight women had to be pooled into one group for the lack of obese women. Nevertheless, the results of Ness et al. are in line with our supplementary data indicating that the inverse association decreases as women’s BMI increases, although lack of statistical power in our data impedes conclusive results on smoking as an effect modifier.

### Study strengths and limitations

The present study was conducted over a 15-year period in Iceland, where women have equal access to prenatal care free of charge [12]. The data originated from women who gave birth at the National University Hospital, where over 75% of all births in the country occurred [13]. The study strengths mainly lie in the homogeneous study population, uniform diagnostic- and health care system, which reduce residual confounding by factors such as socio-economic status and differential prenatal care. Although the biological effects studied are likely to be generalizable beyond the Icelandic population, this cannot be empirically validated and, as such, the homogeneity may compromise the generalizability of our findings.

Our study has several limitations. First, we restricted the study population to women with no registered diagnosis of diabetes, as screening was not uniform during the study period. Future work will need to be directed at understanding the role of diabetes as a confounder/mediator of the association between BMI and hypertensive disorders, and the way in which it impacts on the observed interaction between BMI, smoking status, and hypertensive disorders. Second, the exact dates of hypertensive diagnoses were not available, limiting our possibility to know if the disorder had an early or late onset (i.e. before or after week 34 of pregnancy). Previous findings have indicated that while early onset of preeclampsia may be linked to abnormal placentation [34] and have a genetic component [35], late onset is more likely to be related to maternal factors [34, 36]. In both instances, though, obesity has been indicated as a crucial risk factor [21]. Third, although all hypertensive diagnoses in this study were based on ICD-10 codes, misclassification of disease status may be a possibility and our data do not include information on patients’ history or family history of hypertension during pregnancy. To evaluate the validity of diagnoses in the Medical Birth Registry we compared women’s diagnosis status with their mean blood pressure values, as registered in maternity records at the first and last prenatal visits. When we restricted cases to women who additionally met the blood pressure criteria for hypertension according to maternity records, the effect estimates were intensified for both preeclampsia and gestational hypertension. Fourth, misclassification of exposure is also a possibility. To minimize the effect misclassification due to weight gained during early pregnancy, we restricted all analyses to women who had their BMI measured before the end of pregnancy week 15 and found very similar effect estimates as observed in the main analyses. Further, demographic and pregnancy characteristics were similarly distributed between women with known and unknown BMI. Finally, as smoking was self-reported some women may have misreported their status. We would expect such misreporting mainly to have been smokers classified as non-smokers, which would in turn have attenuated the observed difference in effect estimates for hypertensive disorders with overweight/obesity between smokers and non-smokers. Similarly, such misreporting would have attenuated the inverse associations found between smoking and hypertensive disorders.

## Conclusion

In conclusion, our data indicate that obesity and overweight play a crucial role in relation to hypertensive disorders during pregnancy and that smoking may potentially magnify this relation even further. Further research is needed to draw definite conclusions about the combined effects of these lifestyle-related factors on the hypertensive disorders, especially in light of current and previous findings showing that smoking has an independent inverse association with hypertensive disorders during pregnancy.

## Supporting Information

S1 Table Characteristics of Women with and without Information on Body Mass Index during early pregnancy. Abbreviations: BMI, body mass index; p-value, Fisher Exact Test for difference in proportions between BMI availability.
(DOCX)

S2 Table Association of Smoking with Hypertensive Disorders during Pregnancy Stratified by BMI. Abbreviations: BMI, body mass index; CI, confidence interval; HTD, hypertensive disorder of pregnancy; OR, odds ratio. * Adjusted for parity, multiple gestation and maternal age. Cases and controls gave birth in the same calendar year. All cases and controls are restricted to women without any registered diabetes during pregnancy.
(DOCX)

S1 Fig Mean Blood Pressure (mm Hg) by Diagnosis of Hypertensive Disorder during Pregnancy. (A) Controls (no hypertensive disorder during pregnancy) vs. cases (any hypertensive disorder during pregnancy). (B) Controls (no hypertensive disorder during pregnancy) vs. cases (pre-existing hypertension, gestational hypertension or preeclampsia). Abbreviations: SBP, systolic blood pressure; DPB, diastolic blood pressure. *Mean values of mm Hg are adjusted for parity, multiple gestation and maternal age in with linear regression models. Beta values demonstrate the adjusted the difference of mean mmHg value by diagnosis.
(DOCX)
