# Supplementary material for: Body Mass Index, Smoking and Hypertensive Disorders during Pregnancy: A Population Based Case-Control Study
Source: PLoS One. 2016 Mar 24;11(3):e0152187. doi: 10.1371/journal.pone.0152187 (PMC4807030; doi:10.1371/journal.pone.0152187)
Supplement: S1 Table — Abbreviations: BMI, body mass index; p-value, Fisher Exact Test for difference in proportions between BMI availability. (DOCX) [file pone.0152187.s001.docx]

**Supplementary material**

**S1 Table.** Characteristics of Women with and without Information on Body Mass Index during early pregnancy

|  | **Total  N (%)** | **Missing values n (%)** | **BMI available  n (%)** | **p-values** |
| --- | --- | --- | --- | --- |
| Total | 1497 (100) | 52 (100) | 1445 (100) |  |
| **Hypertension** |  |  |  | 0.07 |
| No | 996 (67%) | 34 (65%) | 962 (67%) |  |
| Preexisting | 97 (6%) | 2 (4%) | 95 (7%) |  |
| Gestational | 81 (5%) | 6 (12%) | 75 (5%) |  |
| Preeclampsia | 319 (21%) | 9 (17%) | 310 (21%) |  |
| superimposed | 4 (0%) | 1 (2%) | 3 (0%) |  |
| **Age, years** |  |  |  | 0.067 |
| < 25 | 419 (28%) | 10 (19%) | 409 (28%) |  |
| 25-34 | 820 (55%) | 27 (52%) | 793 (55%) |  |
| ≥ 35 | 258 (17%) | 15 (29%) | 243 (17%) |  |
| **Nationality** |  |  |  | 0.008 |
| Icelandic | 1457 (97%) | 47 (90%) | 1410 (98%) |  |
| other | 37 (3%) | 5 (10%) | 32 (2%) |  |
| missing | 3 (0) | 0 | 3 (0%) |  |
| **Residency** |  |  |  | 0.14 |
| capital area | 1,122 (75%) | 35 (67%) | 1,087 (75%) |  |
| non capital area | 271 (18%) | 14 (27%) | 257 (18%) |  |
| missing | 104 (7%) | 3 (6%) | 101 (7%) |  |
| **Cohabitation** |  |  |  | 1.0 |
| lives with other parent | 1283 (86%) | 45 (87%) | 1238 (95%) |  |
| single | 212 (14%) | 7 (13%) | 205 (5%) |  |
| missing | 2 (0%) | 0 | 2 (0%) |  |
| **Working status** |  |  |  | 0.43 |
| paid work | 1,083 (72%) | 37 (71%) | 1,046 (72%) |  |
| homemarker, not working, disability | 30 (2%) | 2 (4%) | 28 (2%) |  |
| student | 202 (13%) | 5 (10%) | 197 (14%) |  |
| other | 182 (12%) | 8 (15%) | 174 (12%) |  |
| **Parity, n** |  |  |  | 0.084 |
| 0 | 676 (45%) | 17 (33%) | 659 (46%) |  |
| 1 | 452 (30%) | 16 (31%) | 436 (30%) |  |
| ≥ 2 | 369 (25%) | 19 (37%) | 350 (24%) |  |
| **Multiple gestation** |  |  |  | 0.4 |
| No | 1,395 (93%) | 47 (90%) | 1,348 (93%) |  |
| Yes | 102 (7%) | 5 (10%) | 97 (7%) |  |

Abbreviations: BMI, body mass index; p-value, Fisher Exact Test for difference in proportions between BMI availability
