# Supplementary material for: Body Mass Index, Smoking and Hypertensive Disorders during Pregnancy: A Population Based Case-Control Study
Source: PLoS One. 2016 Mar 24;11(3):e0152187. doi: 10.1371/journal.pone.0152187 (PMC4807030; doi:10.1371/journal.pone.0152187)
Supplement: S2 Table — Abbreviations: BMI, body mass index; CI, confidence interval; HTD, hypertensive disorder of pregnancy; OR, odds ratio. * Adjusted for parity, multiple gestation and maternal age. Cases and controls gave birth in the same calendar year. All cases and controls are restricted to women without any registered diabetes during pregnancy. (DOCX) [file pone.0152187.s002.docx]

**S2 Table.** Association of Smoking with Hypertensive Disorders during Pregnancy Stratified by BMI

|  |  |  | **Normal weight** | |  |  | **Overweight** |  |  |  | **Obese** |  |
| --- | --- | --- | --- | --- | --- | --- | --- | --- | --- | --- | --- | --- |
|  | **Total N** | **Cases n (%)** | **Controls n (%)** | **Adjusted OR^*^ (95% Cl)** | **Total N** | **Cases n (%)** | **Controls n (%)** | **Adjusted OR^*^ (95% Cl)** | **Total N** | **Cases n (%)** | **Controls n (%)** | **Adjusted OR^*^ (95% Cl)** |
| **Any HTD** | 817 | 225 | 592 |  | 362 | 138 | 224 |  | 204 | 104 | 100 |  |
| no | 669 | 199 (88.4) | 470 (79.4) | 1 | 289 | 116 (84.1) | 173 (77.2) | 1 | 166 | 87 (83.7) | 79 (79) | 1 |
| yes | 148 | 26 (11.6) | 122 (20.6) | 0.53 (0.33-0.84) | 73 | 22 (15.9) | 51 (22.8) | 0.57 (0.32-1.01) | 38 | 17 (16.3) | 21 (21) | 0.72 (0.34-1.51) |
| **Preeclampsia** | 748 | 156 | 592 |  | 314 | 90 | 224 |  | 155 | 55 | 100 |  |
| no | 607 | 137 (87.8) | 470 (79.4) | 1 | 245 | 72 (80) | 173 (77.2) | 1 | 123 | 44 (80) | 79 (79) | 1 |
| yes | 141 | 19 (12.2) | 122 (20.6) | 0.55 (0.32-0.93) | 69 | 18 (20) | 51 (22.8) | 0.70 (0.37-1.32) | 32 | 11 (20) | 21 (21) | 0.79 (0.33-1.90) |
| **Gestational hypertension** | 627 | 35 | 592 |  | 247 | 23 | 224 |  | 115 | 15 | 100 |  |
| no | 503 | 33 (94.3) | 470 (79.4) | 1 | 193 | 20 (87) | 173 (77.2) | 1 | 93 | 14 (93) | 79 (79) | 1 |
| yes | 124 | 2 (5.7) | 122 (20.6) | 0.24 (0.06-1.02) | 54 | 3 (13) | 51 (22.8) | 0.57 (0.16-2.08) | 22 | 1 (7) | 21 (21) | 0.23 (0.03-1.95) |
| **Pre-existing hypertension** | 626 | 34 | 592 |  | 249 | 25 | 224 |  | 134 | 34 | 100 |  |
| no | 499 | 29 (85.3) | 470 (79.4) | 1 | 197 | 24 (96) | 173 (77.2) | 1 | 108 | 29 (85.3) | 79 (79) | 1 |
| yes | 127 | 5 (14.7) | 122 (20.6) | 0.67 (0.25-1.77) | 52 | 1 (4) | 51 (22.8) | 0.13 (0.02-1.02) | 26 | 5 (14.7) | 21 (21) | 0.75 (0.24-2.33) |

Abbreviations: BMI, body mass index; CI, confidence interval; HTD, hypertensive disorder of pregnancy; OR, odds ratio.

* Adjusted for parity, multiple gestation and maternal age. Cases and controls gave birth in the same calendar year. All cases and controls are restricted to women without any registered diabetes during pregnancy.
