# Supplementary material for: Body Mass Index, Smoking and Hypertensive Disorders during Pregnancy: A Population Based Case-Control Study
Source: PLoS One. 2016 Mar 24;11(3):e0152187. doi: 10.1371/journal.pone.0152187 (PMC4807030; doi:10.1371/journal.pone.0152187)
Supplement: S1 Fig — (A) Controls (no hypertensive disorder during pregnancy) vs. cases (any hypertensive disorder during pregnancy). (B) Controls (no hypertensive disorder during pregnancy) vs. cases (pre-existing hypertension, gestational hypertension or preeclampsia). Abbreviations: SBP, systolic blood pressure; DPB, diastolic blood pressure. *Mean values of mm Hg are adjusted for parity, multiple gestation and maternal age in with linear regression models. Beta values demonstrate the adjusted the difference of mean mmHg value by diagnosis. (DOCX) [file pone.0152187.s003.docx]

**Panel A**


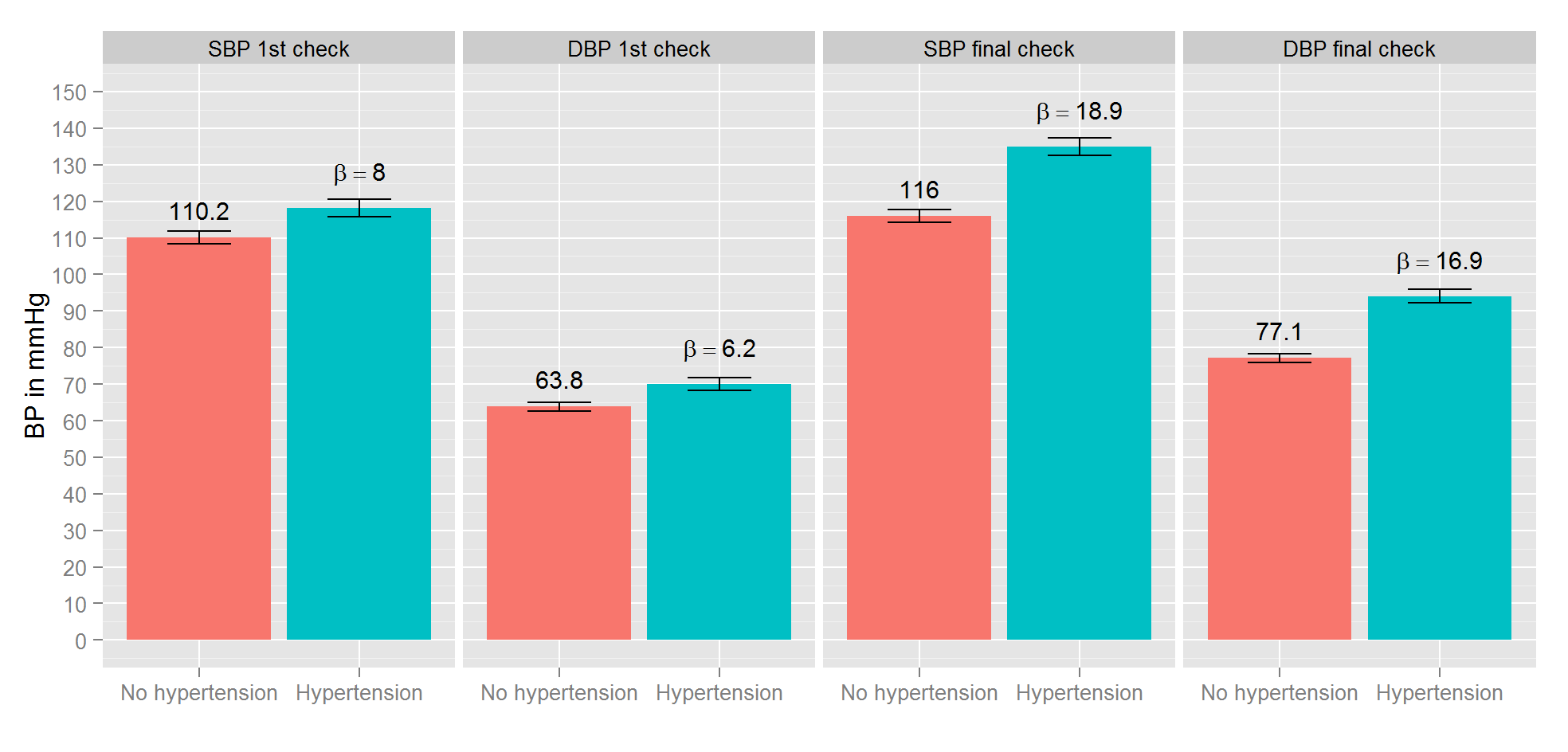


**Panel B**

**
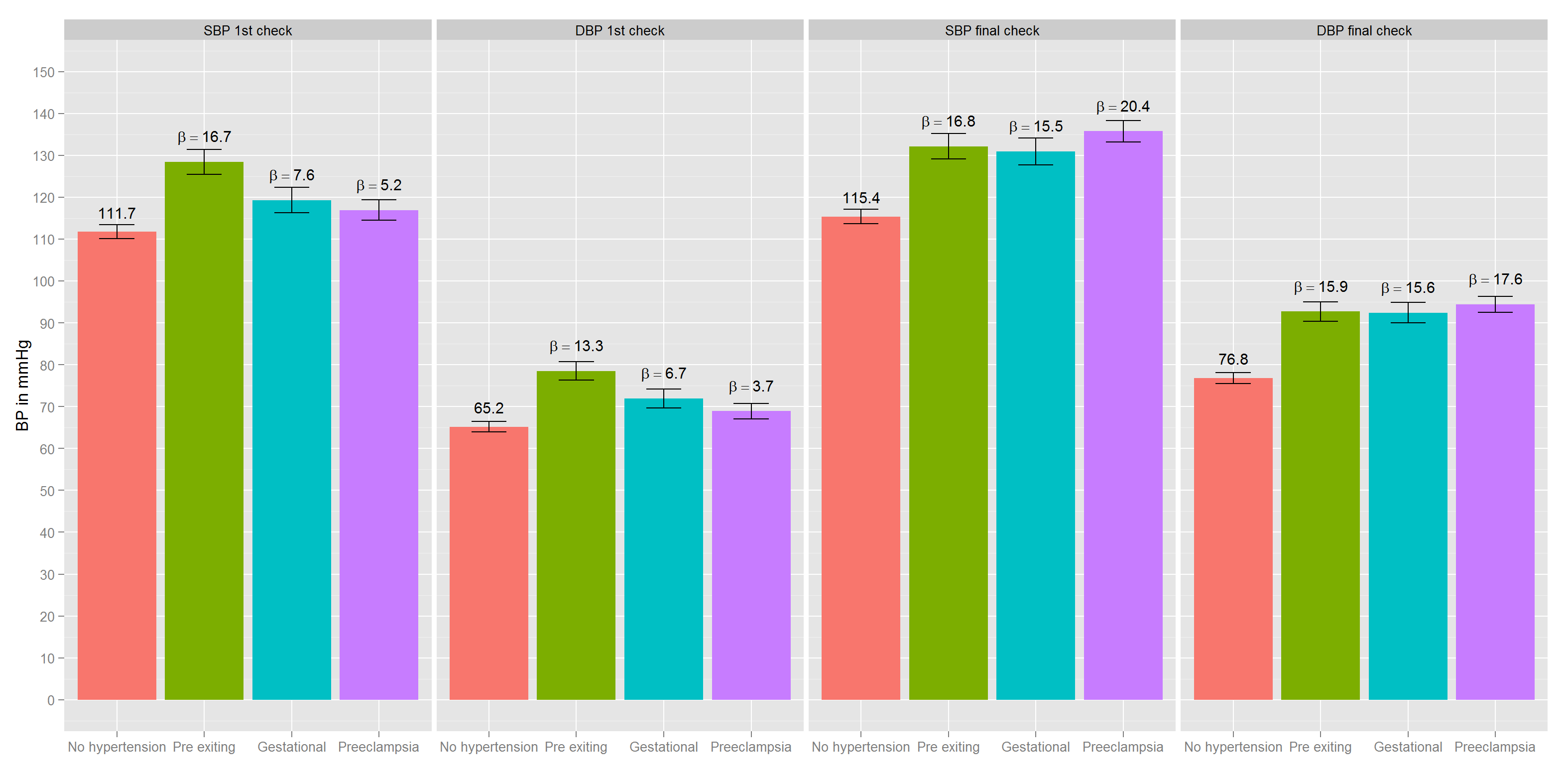
**

**Figure S1.** Mean Blood Pressure (mm Hg) by Diagnosis of Hypertensive Disorder during Pregnancy

(A) Controls (no hypertensive disorder during pregnancy) vs. cases (any hypertensive disorder during pregnancy)

(B) Controls (no hypertensive disorder during pregnancy) vs. cases (pre-existing hypertension, gestational hypertension or preeclampsia)

*Mean values of mm Hg are adjusted for parity, multiple gestation and maternal age in with linear regression models. Beta values demonstrate the adjusted the difference of mean mmHg value by diagnosis.

Abbreviations: SBP, systolic blood pressure; DPB, diastolic blood pressure
